# Prolidase deficiency associated with systemic lupus erythematosus (SLE): single site experience and literature review

**DOI:** 10.1186/1546-0096-10-18

**Published:** 2012-06-22

**Authors:** Yonatan Butbul Aviel, Hana Mandel, Emily Avitan Hersh, Reuven Bergman, Orly Eshach Adiv, Anthony Luder, Riva Brik

**Affiliations:** 1 Department of Pediatrics B, Haifa, Israel; 2 Pediatric Rheumatology service, Haifa, Israel; 3 Meyer Children's Hospital, Rambam Medical Center, Haifa, Israel; 4 Rappaport Faculty of Medicine, Technion-lsrael Institute of Technology, Haifa, Israel; 5 Metabolic Unit, Haifa, Israel; 6 Department of Dermatology, Haifa, Israel; 7 Rambam Medical Center, Haifa, Israel; 8 Pediatric Gastroenterology and Nutrition Unit, Haifa, Israel; 9 Department of Pediatrics and Genetics Service, Ziv Medical Centre, Safed, Israel

## Abstract

**Introduction:**

Prolidase deficiency (PD) is a rare autosomal recessive disorder which may have a wide spectrum of clinical features. These features include a characteristic facies, cognitive impairment, rashes or skin ulceration, splenomegaly, recurrent infections involving mainly the respiratory system, and iminodipeptiduria. The disorder is caused by a mutation in the PEPD gene.

**Objective:**

To describe a cohort of unrelated PD patients from Northern Israel whose inborn error of metabolism was associated with systemic lupus erythematosus (SLE) and to identify in the medical literature all PD cases mimicked by and/or associated with SLE.

**Methods:**

Three patients with PD associated with SLE were clinically, biochemically and genetically investigated. These patients were from 3 unrelated consanguineous families residing in Northern Israel. A computer-assisted (PubMed) search of the medical literature from 1975 to 2011 was performed using the following key words: Prolidase deficiency, SLE, and systemic lupus erythematosus.

**Results:**

An association between PD and SLE was found in 10 PD patients. These 10 patients included three from our cohort of 23 PD patients, and seven out of just under 70 PD patients previously reported in the literature.

**Conclusion:**

The present findings underscore the relatively high incidence of the association between SLE and PD, suggesting that this association may not be coincidental. The phenotypic similarities between SLE and PD might suggest that the PEPD gene constitutes a modifier gene or a genetic risk factor in the causation of SLE.

## Background

Prolidase deficiency (PD) (McKusick 170100) is a rare recessive disorder with an estimated incidence of 1–2 per 1 million births [1]. Prolidase, a ubiquitously distributed dipeptidase, is involved in the latter stages of degradation of endogenous and dietary proteins. It is particularly important in collagen catabolism by affecting the hydrolysis of proline- or hydroxyproline-containing dipeptides at the C-terminal position. The phenotypic expression of PD is highly variable and typically includes mental retardation, recurrent respiratory infections, splenomegaly and dysmorphic facies. A major feature is chronic intractable ulceration of the skin, particularly on the lower limbs. Although the age of onset of the disease varies from birth to 22 years of age, some cases remain asymptomatic [1,2].

Deficiency of prolidase activity leads to massive imidopeptiduria. Various diagnostic techniques have been developed for the detection of imidodipeptides in the urine. However, confirmation of the diagnosis requires the measurement of prolidase activity in erythrocytes, leukocytes or fibroblasts in culture and/or sequence analysis of the PEPD gene [1].

SLE is a multi-system autoimmune disease characterized by widespread inflammation of blood vessels and connective tissues. It has very variable clinical manifestations and specific autoantibodies. The estimated prevalence of SLE in adults is 12–50 per 100,000 individuals [3,4]. Approximately 15-20% of SLE cases occur before the age of 16 years. Onset of SLE is rare before 5 years of age. Pediatric SLE usually results in more aggressive disease and a poorer prognosis [5,6].

An association between prolidase deficiency and systemic lupus erythematosus has been previously reported in the literature [7-10]. Both prolidase deficiency and SLE are associated with disturbances in immune function and have many clinical features in common. It is quite possible that prolidase deficiency is a risk factor for development of SLE. The results of our study underscore the relatively high incidence of the association between SLE and PD and support that this association is not coincidental.

## Case series

### Patient 1

A 4.5 year-old Druze boy, of consanguineous healthy parents, was referred for evaluation of vasculitic skin lesions over his hands, feet, fingertips and earlobes. These lesions had started six months prior to admission. His seven year old brother is healthy. One distant relative was diagnosed with SLE at the age of 28.

**Figure 1:**
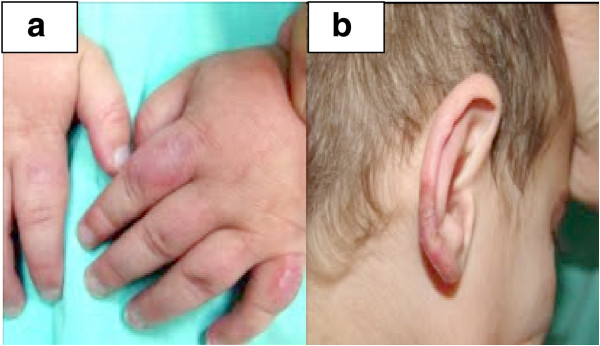
Clinical and histopathological findings of patient 1: a: well demarcated edematous plaques on the dorsal side of the fingers. b: scaly plaques with central atrophy on both earlobes clinically compatible with Discoid Lupus Erythematosus.

At the age of two, a purple rash appeared on the back of his hands, on the tip of all his fingers and his ear lobes. There was no history of fever, arthritis, alopecia, oral lesions, or respiratory or gastrointestinal symptoms. On admission, he appeared well. He had dysmorphic facial features. Vasculitic lesions were observed on the back of his hands, ear lobes, feet and fingertips (Figure 1a - 1b). The rest of the physical examination was normal.

Laboratory findings: Hemoglobin concentration was 12 g/dl, white blood cell count 12,000/μL, platelets 113 × 103/μL, and Westergren erythrocyte sedimentation rate (ESR) 45 mm/h. The CRP was normal. There was mild elevation of liver enzymes. The urinalysis, serum electrolytes, urea, creatinine, creatine kinase, albumin and bilirubin concentration tests were normal.

**Figure 2:**
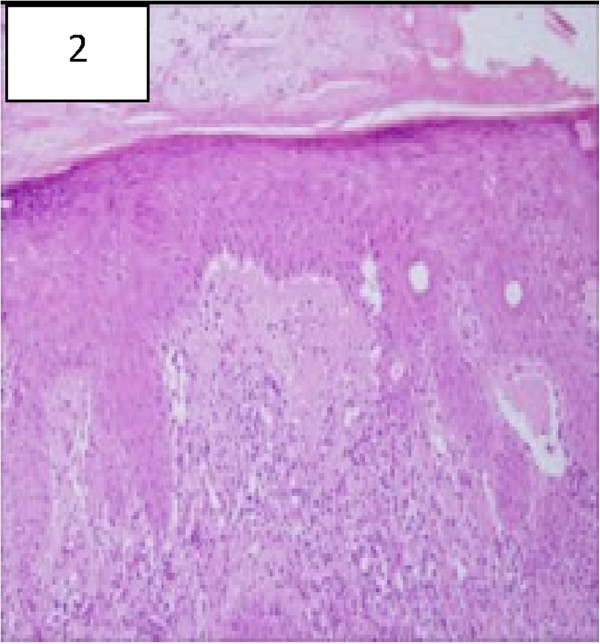
Histopathology of the involved skin from the dorsal aspects of a finger showing typical changes of cutaneous Lupus Erythematosus including vacuolar degeneration of the basal- cell layer, and focal thickening of the basement membrane (hematoxylin and eosin, x100).

An immunological evaluation revealed a high serum concentrations of IgG2 (960 mg/dl) IgA (667 mg/dl),and a normal level of IgM. Antinuclear antibodies (ANA) were positive at a titer of 1:640 (homogenous pattern). The antibodies to double-stranded DNA, Anti-RNP, anti-SM, Anti Ro (SS-A) and Anti La (SS-B) were also elevated. Complement levels of C3 and C4 were low. Antibodies to cardiolipin, SCL-70, PM-1, and centromere were negative. A skin biopsy was compatible with hypertrophic discoid lupus (Figure 2).

Urinary amino acid analysis revealed hyperiminodipeptiduria, which was suggestive of PD. The diagnosis of PD was confirmed by mutation analysis of the PEPD gene, revealing the S202F missense mutation which was previously found to be common among the Druze with PD in Northern Israel and the Golan Heights (10). Treatment consisted of oral prednisone 1 mg/kg/d and hydroxychloroquine 6 mg/kg/d. The child improved well on treatment, except for the discoid skin lesions. At the age of 4.5 years he presented with proteinuria and mild hematuria. A renal biopsy was performed and demonstrated glomerular changes consistent with WHO Class IV Lupus nephritis. High dose prednisone was begun at a dose of 2 mg/kg as well as mycophenolate mofetil at a dose of 1200 mg/m² with gradual improvement.

### Patient 2

A 16-year-old Moslem Arab girl, the second of three healthy children, born to consanguineous healthy parents, was admitted to the hospital at the age of 5 yrs with a lobar pneumonia. There was no family history suggestive of PD. She was hospitalized at the age of 1 month because of a diffuse skin rash and failure to gain weight, which was attributed to milk allergy. A skin biopsy was compatible with seborrheic dermatitis. Local steroid cream applications and hypoallergenic formula feeding were instituted, with an apparent good response. She was admitted again at three months of age with bacterial pneumonia. Evaluation for underlying immunodeficiency syndrome and cystic fibrosis was negative. During the following years the child exhibited mild developmental delay. Splenomegaly was detected as an outpatient.

**Figure 3:**
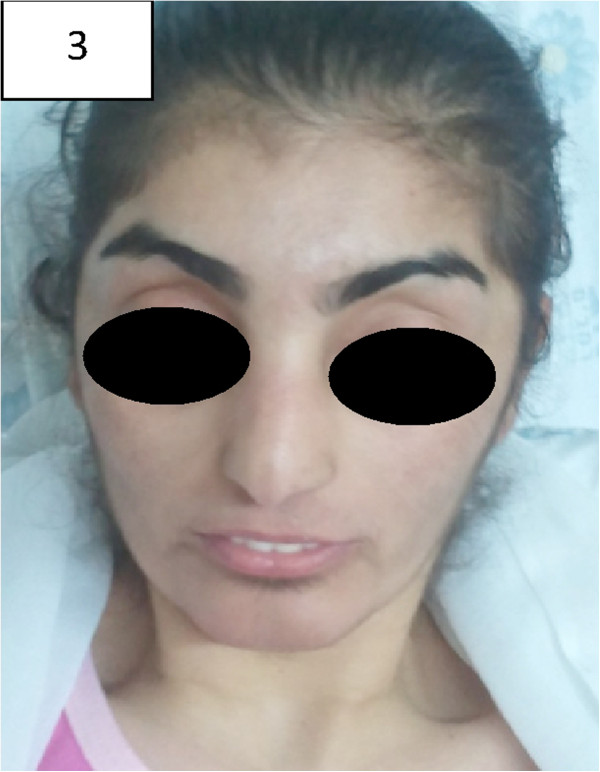
Patient no. 2 depicting the characteristic facial dysmorphism of PD.

On her third admission under 1 year of age, the patient looked thin and dysmorphic (Figure 3). She had microcephaly, a prominent metopic suture, clinodactaly and brachydactyly. There were no skin lesions. The heart and lung examinations were normal. A soft spleen was palpated 6 cm below the left costal margin; the liver was not enlarged. The rest of the physical examination was normal.

Laboratory findings at that time: Hemoglobin concentration of 9 g/dl, white blood cell count 5.1 × 103/μL, and platelets 171 × 103/μL. The following tests were within normal limits: urinalysis, Westergren erythrocyte sedimentation rate (ESR), serum electrolytes, urea, creatinine, creatine kinase, albumin, and liver function tests. Bone marrow aspiration and a gallium scan were also normal. In view of the patient’s dysmorphic features and past clinical history, she underwent a metabolic work-up. The results were consistent with the diagnosis of PD. The diagnosis of PD was confirmed by mutation analysis of the PEPD gene which revealed the A212P missense mutation in exon 2 [10]. The patient was treated with antibiotics and was subsequently followed up by our Metabolic Unit.

Three years later the patient was admitted following seven days of fever, with left lower lobe pneumonia, macroscopic hematuria and proteinuria. Laboratory evaluation demonstrated acute renal failure, pancytopenia and hypoalbuminemia. Immunological studies showed a highly positive antinuclear antibodies assay (ANA), and a positive double stranded DNA titer. There were low levels of C3 and C4. Anti-Ro/SS-A anti La/SS-B, anti-RNP, anti-SM and anti-cardiolipin antibodies were negative by immunodiffusion. A direct Coombs test was positive. A renal biopsy was performed and demonstrated glomerular changes consistent with WHO Class IV Lupus nephritis. Following diagnosis of SLE, treatment with pulse methylprednisolone and pulse cyclophosphamide was begun. Nevertheless, the patient's renal function deteriorated rapidly; she became anuric and required hemodialysis. With continuing medication and dialysis over five months, the renal function improved, and the patient could gradually be weaned off hemodialysis, although massive proteinuria persisted. A few months later she presented with a focal seizure on her left side and hypertension. She underwent further evaluation including MRI of the brain which showed multiple bilateral subcortical white matter lesions mainly over the parietooccipital area with leptomeningeal enhancement. Her lumbar puncture results were normal. The findings were consistent with CNS vasculitis. The patient was treated with pulse methylprednisolone and continued therapy with pulse cyclophosphamide. Her seizures stopped and she recovered completely. Eventually, end-stage renal failure developed in spite of therapy with high dose steroids, cyclophosphamide, azathioprine, and anti-hypertensive drugs. The patient has recently undergone successful renal transplantation. Her lupus disease has been inactive for the last year on only a low dose of daily steroids.

### Patient 3

A 24-year-old Druze female is the seventh child of 1st degree consanguineous, healthy Parents. Her paternal uncle was diagnosed with PD and died of severe lung disease a few years prior to this patient’s first admission. She had been followed in another hospital since birth. She was noted to have mild developmental delay and recurrent episodes of bacterial pneumonia. Over the years she also developed severe clubbing of the fingers, bronchiectasis, a non-specific maculopapular rash of the skin, splenomegaly, and pancytopenia. At 12 years of age she was referred to our hospital for further evaluation.

**Figure 4:**
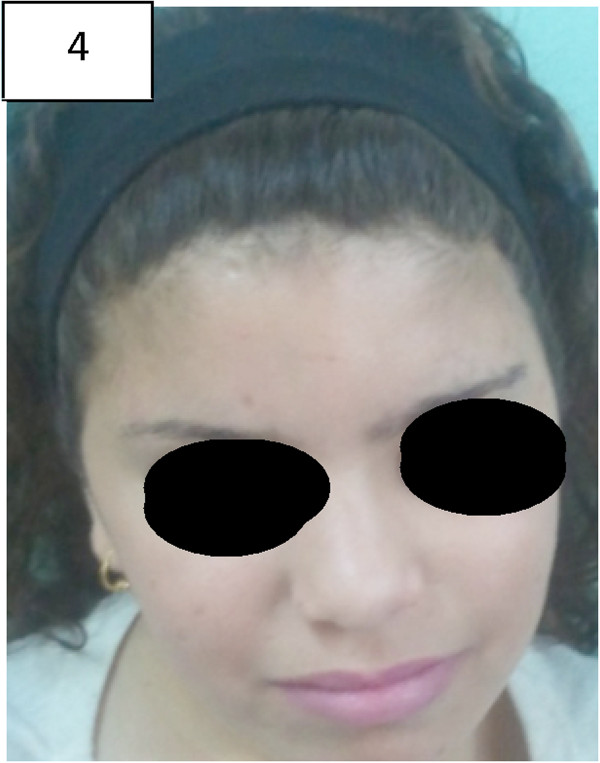
Patient no. 3 with milder dysmorphic features.

On admission, her characteristic dysmorphic features (Figure 4) reminded us of our previous patient with prolidase deficiency. The diagnosis of PD in this patient was confirmed by metabolic enzymatic studies and by mutation analysis of the PEPD gene which revealed the S202F missense mutation similar to the first patient [10]. In addition, a diagnosis of concurrent SLE was made on the basis of the following hematological and immunological studies: thrombocytopenia, positive Coombs test, positive ANA, positive dsDNA and low C3 and C4. Other antibodies titers including anti-cardiolipin and lupus anti- coagulants were also positive. Anti Ro/SS-A, anti La/SS-B, anti Sm were negative. The urine analysis was repeatedly normal.

The patient was treated for severe thrombocytopenia with high dose steroids, IV- immunoglobulin, and cyclophosphamide. Yet she continued to have thrombocytopenia, thus necessitating splenectomy. The hematological and immunological manifestations of SLE resolved nine months after she had the splenectomy. Currently, twelve years after the initial diagnosis of SLE, the patient is on low dose prednisone maintenance therapy mainly for mild arthritis and progressive lung disease. CT imaging of the chest demonstrated mainly cystic lung lesions and ground glass opacity. Recently she experienced further deterioration in her pulmonary function and secondary pulmonary hypertension. At present she is oxygen dependent.

## Discussion

Prolidase deficiency (PD) (OMIM 170100) is a rare disease with an estimated incidence of 1–2 cases per million births. This disease is probably underdiagnosed. (1). This autosomal recessive, pan-ethnic disorder is characterized by a highly variable phenotype including, rashes or skin ulceration, dysmorphic features, cognitive impairment, anemia, splenomegaly, and recurrent infections. Fewer than 70 PD patients have been documented in the medical literature, 10 of them were reported to have SLE features. Twenty-three patients with PD are currently followed in our institution of which only the above 3 patients have developed SLE (13%), This association is much higher than the association of SLE in the normal population.

The prolidase enzyme hydrolyzes dipeptides that have either carboxy-terminal proline or Hydroxyproline. Prolidase has a major role in the recycling of proline released during the degradation of collagen and dietary proteins [11]. Although there is considerable knowledge concerning the putative roles of the prolidase enzyme [12], the pathophysiology of PD remains an enigma.

Prolidase is particularly important in collagen catabolism. Cultured fibroblasts from patients with PD show changes that might be associated with a necrosis-like cellular death [13]. This change may be related to the intracellular accumulation of the Gly-Pro dipeptides which could be responsible for the typical skin lesions in PD, as well as for the association with SLE caused by exposure to intracellular protein. Ultrastructurally, collagen molecules have been found to be shortened and to show non-homogeneous packing of fibrils [14]. Thus it appears that the pathogenesis may involve angiopathy of small blood vessels, probably due to disturbance of this connective tissue metabolism, caused by deficiency of the prolidase enzyme [15]. Another mechanism that connects PD with lupus might be through complement components such as C1q, which has high proline content. Hypothetically, PD may cause the synthesis of antigenically immunoreactive, but biologically defective, C1q, that alters immune responses and thus predisposes to autoimmune disease (9). This association between SLE and prolidase deficiency has been reported previously in 7 cases (2 of our cases patient number 2 and 3 were previously briefly reported as part of a large series of PD patients) [7-10,16]. The clinical features and laboratory findings are presented in Table 1.

**Table 1:** Clinical and laboratory findings in SLE associated with prolidase deficiency

**Patient no (Reference)**	**1 Shrinath (1997**^**3**^**)**	**2 Shrinath (1997**^**3**^**)**	**3 Klar (2009)**	**4 Klar (2009)**	5 Rocco (2007*^4^**)**	**6 Bisonnette (1993**^**9**^**)**	**7 Marotte (2010)**^**1**^	**8 Falik-Zaccai (2010**^**10**^**)**	**9 Falik-Zaccai (2010**^**10**^**)**	**10 Present report**
**Consanguinity**	+	+	+	+	?	?	unknown	+	+	+
**Ethnicity**	Pakistan	Pakistan	Arab Moslem	Arab Moslem	?	?	Portugal	Druze	Arab Moslem	Druze
**Sex**	M	F	F	M	M	F	M	F	F	M
**Age of PD/SLE diagnosis**	9m/8y	6m/3y	/8y	/12y	?	/16y	/25y		**5/8**	**2y/2.5**
**Skin involvement**	+	+	+	+	+	+	+	+	+	+
Maculo-papular rash	+	+	+	-	+	+	-	+	+	+
Vasculitis	+	-	-	-	**+**	-	Necrotic lesion	-	-	**+**
Leg ulcers	-	-	**+**	**+**	**+**	**+**	**+**	-	-	-
**Splenomegaly**	**+**	-	**+**	**+**	-	-	**+**	**+**	**+**	**+**
Splenectomy	-	-	-	-		-		**+**	-	-
**CNS involvement**	-	-	-	-	-	-	-	**_**	**+**	**_**
seizure									**+**	
**Renal disease**	+	-	-	-	-	+	+	-	+	+
Kidney biopsy	-	-	-			Class III	+		Class IV Kidney transplantation	**Class IV**
Renal failure	-		-			-				**-**
**Hematological Disease**	+	+	+					+	-	+
Anemia/Leukopenia/thrombopenia	+	+	+					+	-	+
**Recurrent infections**	+	+	+	+	-	+		+	+	-
**Pulmonary disease**	+	+	+	+		-	+	+	+	-
ANA	+	+	+	-	+	+		+	+	+
**Positive ENA**	+	+	+	+	+	+	1/640	+	+	+
Anti DNA	+	+	+	+	+	+	-	+	+	+
Hypocomplementemia	+	-	-	+	+	+	+	+	+	+
**Fulfilled Criteria of SLE**	+	+	-	-	-	+	"Rhupus"^**2**^syndrome	+	+	+

Most of the PD patients reported to have SLE present relatively early in childhood, (average age 8.3 ± 4.6 years). The most common presenting symptoms in combined PD and SLE are recurrent infections and variable skin lesions. Leg ulcers were a predominant feature and appeared in 5/10 patients. This clinical feature contrasts with patients who have SLE without PD, in whom leg ulcers are rarely reported. Lung disease among patients with PD and SLE was found in 7/10 cases. Most of the reported cases were bronchiectasis that may be due to the recurrent infections. In one of our cases (no. 3), the main lung disease was of cystic lung lesions combined with ground glass appearance that may be due to PD and less likely due to SLE. In contrast, clinical and subclinical pleuropulmonary disease is a frequent manifestation of childhood SLE. The most frequently reported and described pleuropulmonary manifestations are pleural effusion, pleuritis, acute and chronic pneumonitis, and pulmonary hemorrhage [17-19]. Acute lupus pneumonitis consisting of pulmonary infiltrates and atelectasis, occurs in 10–15% of children with SLE. Chronic interstitial lung disease has been reported in adults with SLE [17,20], but not in children. Cystic lesions similar to the finding in the imaging of patient no. 3 are not characteristic of SLE and are more likely related to PD. Recently there have been a few reports of patients with prolidase deficiency without SLE who developed cystic lung lesion [21,22]. In reported cases of PD patients that developed SLE, most patients had some response to steroids and immunosuppressive therapy; however skin lesions were usually unresponsive to corticosteroid treatment. There were a few reports of mortalities in these patients, most of them related to infections [7-10].

## Conclusions

There appears to be an association between prolidase deficiency and SLE. Awareness of this observation may be important in furthering our understanding of the etiology of SLE. We recommend that an underlying diagnosis of PD should be considered in some patients with SLE, especially in patients who present with dysmorphic features, developmental delay and poorly responsive skin disease, as well as in patients who have SLE and recurrent lung infections resembling cystic fibrosis. We also believe that patients with an established diagnosis of PD should be regularly monitored for an underlying autoimmune disorder.

## Consent

"Written informed consent was obtained from the patients for publication of these case reports and any accompanying images. A copy of the written consent is available for review by the Editor-in-Chief of this journal."

## Abbreviations

PD: Prolidase deficiency; SLE: Systemic lupus erythematosus; ESR: Erythrocyte sedimentation rate.

## Competing interests

The authors declare that they have no competing interests.

## Authors' contributions

BY participated in the sequence alignment and drafted the manuscript, MH helped to draft the manuscript, AE helped to draft the manuscript, BR helped to draft the manuscript, EO helped to draft the manuscript, LA helped to draft the manuscript, BR helped to draft the manuscript. All authors read and approved the final manuscript.
